# New insights into the morphogenic role of stromal cells and their relevance for regenerative medicine. lessons from the heart

**DOI:** 10.1111/jcmm.12247

**Published:** 2014-02-18

**Authors:** Daniele Bani, Silvia Nistri

**Affiliations:** Department of Experimental & Clinical Medicine, Section of Anatomy & Histology, Research Unit of Histology & Embryology, University of FlorenceFlorence, Italy

**Keywords:** stromal cells, extracellular matrix, morphogenesis, regeneration, repair, scarring, fibroblasts, telocytes

## Abstract

The term stromal cells is referred to cells of direct or indirect (hematopoietic) mesenchymal origin, and encompasses different cell populations residing in the connective tissue, which share the ability to produce the macromolecular components of the extracellular matrix and to organize them in the correct spatial assembly. In physiological conditions, stromal cells are provided with the unique ability to shape a proper three-dimensional scaffold and stimulate the growth and differentiation of parenchymal precursors to give rise to tissues and organs. Thus, stromal cells have an essential function in the regulation of organ morphogenesis and regeneration. In pathological conditions, under the influence of local pro-inflammatory mediators, stromal cells can be prompted to differentiate into myofibroblasts, which rather express a fibrogenic phenotype required for prompt deposition of reparatory scar tissue. Indeed, scarring may be interpreted as an emergency healing response to injury typical of evolved animals, like mammals, conceivably directed to preserve survival at the expense of function. However, under appropriate conditions, the original ability of stromal cells to orchestrate organ regeneration, which is typical of some lower vertebrates and mammalian embryos, can be resumed. These concepts underline the importance of expanding the knowledge on the biological properties of stromal cells and their role as key regulators of the three-dimensional architecture of the organs in view of the refinement of the therapeutic protocols of regenerative medicine.

## Importance of correct three-dimensional organization of multicellular entities

Cell biologists familiar with *in vitro* cell cultures are well aware of the fact that, when removed from their tissue source and adapted to *in vitro* conditions, eukariotic cells behave as semi-amorphous organisms, capable of crawling and mutually adhering to give rise to tissue-like layers or masses, but substantially unable to form evolved patterns of tissues and organs, although they possess all the needed genes. In relatively limited instances, under the influence of appropriate microenvironmental signals, cultured cells can re-create a tissue architecture resembling that of the original tissue, as occurs for autologous epidermal layers used for grafting purposes in burned patients [1]. The mechanisms controlling the three-dimensional assembly of cells to give rise to differentiated tissues and organs are a crucial issue in stem cell biology as well as regenerative medicine. As a matter of fact, the odds of favourable outcome of stem cell grafting for organ repair are reduced when the host organ has a complex three-dimensional architecture, and even more inconsistent when this architecture has been altered by pathological processes. At present, widespread use of stem cell grafting for clinical purposes is only available for non-coherent organs, such as the bone marrow, which can be effectively and completely replaced by transplantation of hematopoietic stem cells. On the other hand, stem cell–based treatment of the failing heart can be assumed as a typical example of the substantial inability of the regenerative approach to re-create a structurally complex tissue such as the myocardium [2].

## Role of extracellular matrix in morphogenesis

The embryonic development offers us a clear paradigm of the events and mechanisms that come into play to allow the transition from undifferentiated rudiments to well-defined organ precursors. These events are basically characterized by the appearance of the mesenchyme and, soon after, mesenchyme-derived extracellular matrix (ECM). The functions of ECM in the embryo are numerous, but can be resumed in the concept that ECM is capable of forming rigid, semirigid and plastic structures perfectly adapted to integrate cells into functional assemblies and regulate their differentiation, thereby determining the proper shape of the organs and the whole body. In turn, differentiating cells are tuned to make the proper ECM molecules (collagens, proteoglycans and other matrix proteins) and may switch the type of matrix molecules they produce to meet the requirements of time and place [3]. Moreover, by these same matrix molecules, ECM can provide feedback information to cells, a mechanism that contributes to embryonic induction [4]. In a typical paradigm, spatiotemporal deposition of ECM components, such as fibronectin, has been reported to influence the correct migration of myocardial precursor cells to form the primitive heart tube [5]. What is understood is that ECM molecules can affect the organization of the cytoplasm *via* surface receptors and thereby influence the shape, mobility and differentiation of the cell [3, 6–8]. Indeed, the classical concept that spatially oriented changes in cell growth, migration and differentiation are mediated by soluble factors has been flanked by the notion that mechanical forces contribute to morphogenesis at the same extent as the soluble molecules [9]. In particular, through transmembrane receptors, ECM and cell cytoskeleton are linked in an interconnected system capable of generating and sensing the tensional forces occurring in the tissue. These physical stimuli can modify cellular signalling, thereby switching the cell fate [10]. In this context, mechanical signals generated by haemodynamic stresses in the beating primitive heart tube have been shown to play a key role in heart compartmentalization and valve formation [11]. Similarly, the mechanical characteristics of the ECM can regulate self-renewal and lineage differentiation of stem cells. For instance, mesenchymal precursors can give rise to neuronal-like cells if grown on soft ECM, to osteoblasts on stiff ECM and to myoblasts on ECM with intermediate stiffness [12].

A bulk of studies have addressed the morphogenetic role of ECM, but this goes beyond the specific object of the present article; the reader is referred to previous, authoritative reviews for a full discussion of this matter [3, 6–10].

## Role of stromal cells in morphogenesis

Mesenchymal cells are the first type of stromal cells that appear during embryonic development. At variance with the cells of the embryonic sheets, such as the ectoderm and endoderm, these cells do not express the cell–cell adhesion complexes required for epithelial cohesiveness, thus becoming mobile. Like mature fibroblasts, they synthesize the macromolecules that compose the ECM in which they reside. Upon further development, stromal cells with their ECM accompany every tissue and organ. This connective tissue, or stroma, is of pivotal importance for the final architecture and function of organs in the mature organism [13, 14]. Of note, the many different shapes of differentiated tissues and organs are an expression of the shape of the ECM, which in turn was determined by stromal cells responsible for moulding it. Using the appropriate words of Doljanski [13], ‘it can be generalized that the ECM is the biological entity that expresses morphology, and that (stromal) cells are the sculptors that mould the ECM into the appropriate forms'.

The above notions underscore the primary role of mesenchyme-derived cells in determining tissue and organ architecture. This is a rapidly expanding field for biomedical research because of its obvious implications for regenerative medicine. The growing knowledge in this area has also put into evidence that stromal cells have heterogeneous origin and features, as will be briefly sketched in the following chapter.

## Different origin of stromal cell populations

The classical view of stromal cells as mature offspring of mesenchymal precursors has been recently challenged by new knowledge emerged from numerous studies on normal and pathological conditions. Accordingly, stromal cells can be ascribed to three different subsets: real mesenchymal, haemopoietic-derived and arisen from epithelial–mesenchymal transition (EMT) [15]. Unfortunately, because of the limited knowledge of their specific markers, the exact role of each subset in the formation of stroma is neither clearly distinguishable nor understood. A scheme of the recognized stromal cell lineages in the heart and their main distinctive markers is given in Table 1. Moreover, some information comes from studies concerning pathological ECM formation in diseased organs, as occurs during fibrosis, and it is not clear whether the same notions can be applied to physiological conditions. In spite of these limitations, it is generally accepted that all subsets can produce ECM as well as growth/differentiation factors required for the build-up of stroma.

**Table 1:** Diverse cardiac stromal cell lineages and their main markers [15, 47]

**Stem cell**	**Early markers**	**Mature stromal cell**	**Late markers**
Mesenchymal	CD13 CD29 CD44 CD73 CD90 CD105 CD146 Stro-1 PDGFr	Fibroblast	Type I collagen
		Telocyte (?)	CD34 CD117/c-kit
		Adipocyte	PPARγ2 Leptin Adiponectin
		Endothelial cell	CD31/PECAM-1
Haemopoietic	CD45 CD11b	Fibroblast (fibrocyte)	CD45 CD11b Type I collagen CD13 CD29 CD34
		Myofibroblast	α-SMA Type I collagen
		Adipocyte	PPARγ2 Leptin Adiponectin
		Endothelial cell	CD31/PECAM-1
EMT-derived	CD44 Vimentin Fibronectin n-cadherin	Fibroblast	Type I collagen
		Adipocyte (?)	PPARγ2 Leptin Adiponectin

(1) Mesenchymal stromal cells are consistent with the traditional notion of mature cells derived from mesenchymal stem cells permanently residing in adult connective tissues, mainly in perivascular niches [16, 17]. These latter cells are defined mainly by functional assays in *in vitro* culture, where they display fibroblast-like features and express numerous cell surface molecules, including Stro-1, PDGFr, CD13, CD29, CD44, CD73, CD90, CD105 and CD146, while lacking the markers of haematopoietic and endothelial lineages CD45 and CD31/PECAM-1 respectively [18]. Differentiation of mesenchymal progenitors into fibroblasts is proposed to be a major source of stromal cells in both normal development and fibrotic diseases [18]. However, the mesenchymal lineage can also differentiate into chondroblasts, osteoblasts and adipocytes [19] and may serve as pericytes in the vascular wall [20]. Because of the lack of reliable differentiation markers, the borderline between stem and mature mesenchymal stromal cells is currently ill-defined. It has been reported that, under non-physiological circumstances such as hypoxia and inflammation, mesenchymal stem/stromal cells can be mobilized to the bloodstream [21], thereby potentially contributing to stromal cell trafficking between different connective tissue sites.

(2) Haemopoietic-derived stromal cells have been first identified in long-term or starved leucocyte cultures as fibroblast-like cells that retain haematopoietic and leucocytic markers (CD45, CD11b) and are capable of secreting ECM components such as type I collagen [22]. They are thought to arise from a subset of circulating monocyte-like cells [22, 23] and, being blood-borne mobile cells, can substantially contribute to the stromal cell population of connective tissues [24], especially in pathological conditions [25]. Of note, haemopoietic-derived stromal cells have been postulated to give rise to myofibroblasts, the major stromal cell type involved in wound healing and fibrotic diseases [26]. Similar to mesenchymal stromal cells, they can also give rise to adipocytes [25, 27].

(3) Epithelial–mesenchymal transition–derived stromal cells are originated through a peculiar morphogenetic mechanism, originally described during embryo development, whereby epithelial cells lose stable cell–cell and cell–basal lamina junctions and acquire the capability of migrating into the ECM [28]. This is not a mere effect of down-regulation of attachment molecules expressed at the plasma membrane, but involves a broader genomic reprogramming that leads the cells to acquire a true mesenchymal differentiation, as judged by *de novo* expression of vimentin, fibronectin and N-cadherin [29]. It is currently believed that EMT is a typical stemness trait. In fact, EMT-derived stromal cells share with mesenchymal and haemopoietic stromal cell precursors the capability of differentiate into multiple mesenchymal lineages, such as chondroblasts, osteoblast and adipocytes [30]. Although EMT definitely plays an essential role during embryonic development [31], its actual contribution to the normal stromal cell population of adult organs is unknown. On the other hand, EMT can be induced in pathological conditions, such as wound healing, fibrosis and tumorigenesis [15].

## Morpho-functional features of stromal cells in the heart and their possible roles

As stated in the introductory chapter, the heart is a paradigm of organ provided with unique three-dimensional tissue architecture that is moulded during complex organogenesis processes and is crucial to the heart's physiological function [32]. The cardiac stroma plays a critical role in the maintenance of the architecture of the heart, as well as in its pathophysiological alterations occurring in cardiac diseases, such as for instance post-infarction remodelling and cardiac fibrosis [33].

**Figure 1:**
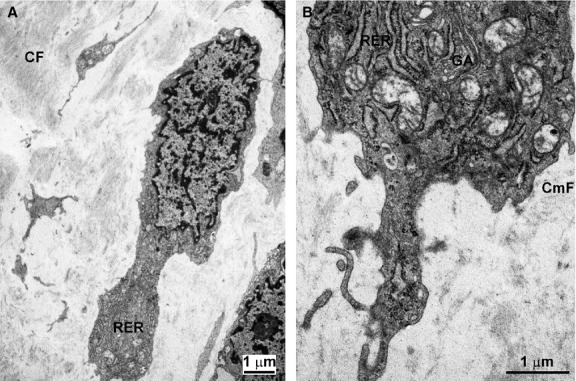
Ultrastructural features of a typical cardiac fibroblast in the swine heart epicardium (A) and a high-magnification detail (B). This cell has elongated shape, euchromatic nucleus and cytoplasm containing profiles of rough endoplasmic reticulum (RER) and Golgi apparatus (GA). The extracellular matrix contains collagen fibres (CF). Collagen microfibrils (CmF) can be seen in the proximity of the cell surface.

Cardiac fibroblasts are the most prominent and best known cardiac stromal cell type that is held responsible for the formation and renewal of ECM. Being differentiated cells, in the normal adult heart their proliferative attitude is very low and presumed to be barely capable to fulfil the need for self-renewal [32]. This property is reflected in the observation that isolated cardiac fibroblasts grow very slowly in culture and rapidly undergo senescence [34]. Morphologically, typical fibroblasts can be identified in the cardiac stroma by transmission electron microscopy. They appear as fusiform or stellate cells with euchromatic nuclei and cytoplasms containing numerous RER profiles and a well-developed Golgi apparatus. Their cell surface lacks a basal lamina and often forms grooves containing thin bundles of collagen microfibrils, indicating that these cells can preside over the spatial orientation of the newly formed ECM macromolecules (Fig. 1). Besides being primarily responsible for ECM production and remodelling, cardiac fibroblasts can also regulate cardiomyocyte proliferation and growth during development through paracrine and juxtacrine signals [35, 36]. Thus, they are currently viewed as a dynamic, multi-functional lineage crucial for both developmental and post-natal repair pathways.

**Figure 2:**
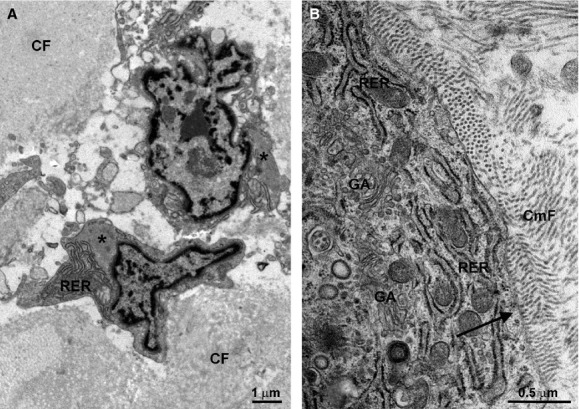
Ultrastructural features of typical myofibroblasts in the ventricular scar of a post-infarcted swine heart (A). These cells show stellate shape, euchromatic nucleus and cytoplasm containing several cisternae of rough endoplasmic reticulum (RER) and bundles of contractile microfilaments (asterisks). Coarse collagen fibres (CF) adhere to the cell surface. (B) Detail of the peripheral cytoplasm of a myofibroblast showing numerous RER cisternae and an extended Golgi apparatus (GA). Collagen microfibrils (CmF) are adjacent to the plasma membrane and appear to be undergoing assembly from tropocollagen monomeres.

In keeping with the notions highlighted in the previous chapter, in pathological conditions, the stromal cell population of the heart can be increased by the contribution of CD45+ haemopoietic-derived precursors, which are recruited by the injured venular endothelium through the release of cytokines, such as monocyte chemoattractant protein 1 (MCP-1) [38, 39]. This mechanism is thought to give rise to the majority of reactive myofibroblasts involved in myocardial interstitial fibrosis [40]. According to this view, resident mesenchymal-derived fibroblasts are involved in reactive scarring, whereas immigrated haemopoietic-derived (myo)fibroblasts are held responsible for adverse myocardial remodelling, characterized by excessive collagen formation, muscle fibre entrapment, muscle atrophy, electrophysiological abnormalities and, most commonly, abnormal cardiac function resulting from increased ventricular stiffness and arrhythmias [32]. Cardiac myofibroblasts are usually stellate cells with euchromatic nuclei and abundant cytoplasms in which RER cisternae and Golgi apparatus co-exist with microfilament bundles. Their surface grooves contain coarse collagen bundles (Fig. 2).

The actual contribution of EMT to the adult population of cardiac stromal cells in health and disease is poorly understood and matter of investigation. This possibility is deemed plausible by similarity with the mammalian embryo, in which cardiac fibroblasts are thought to originate from the epithelial-like pro-epicardium *via* EMT [41, 42]. In case of heart injury, a specific variant of EMT involving the coronary vessels, termed endothelial-to-mesenchymal transition, has been reported to take place and give rise to myofibroblasts [43].

**Figure 3:**
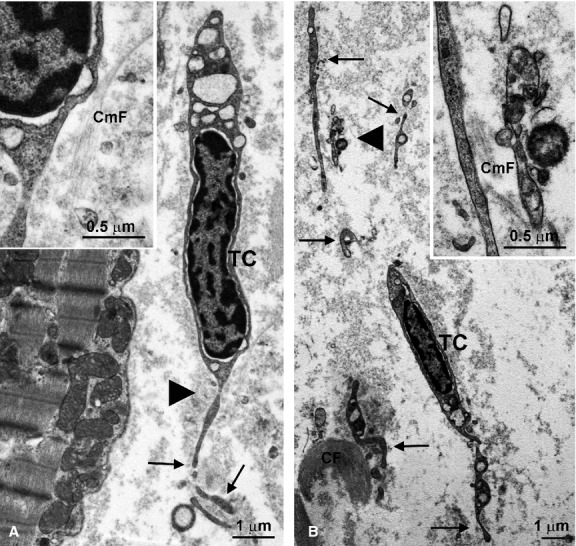
Ultrastructural features of typical telocytes (TC) in the swine heart interstitial stroma (A and B). These cells show spindle-like shape, heterochromatic nuclei and scarce cytoplasms containing large cisternae of rough endoplasmic reticulum. They are provided with extremely elongated telopodes (arrows). Collagen fibres (CF) and microfibrils (CmF) are adjacent to the telopodes (B). The insets show higher magnifications of the areas indicated by the arrowheads.

In most recent years, a new peculiar stromal cell type has been described and characterized in several tissues and organs, including the heart. This has been the fortunate discovery of a talented group of morphologists in Bucharest, Romania, led by L.M. Popescu, who were studying the distribution in the body of interstitial cells of Cajal (ICC), a stellate cell typically interposed between sympathetic nerve endings and smooth muscle cells of the gastrointestinal tract. Authentic ICC are thought to have a smooth muscular origin and to be specifically differentiated to exert pace maker function and mediate the neural control of visceral motility [44, 45]. While searching for ICC in organs other than the gastrointestinal musculature, including the pancreas, male and female reproductive tracts, gallbladder, blood vessels and heart, Popescu and coworkers noticed a cell population located in the interstitial stroma that was roughly similar to ICC and expressed mesenchymal/haemopoietic lineage markers such as CD34, CD117 and cKit. Their widespread distribution in the interstitium of embryologically different organs led to hypothesize that these cells could be a new stromal cell type. Formerly designated with the ambiguous term ‘interstitial Cajal-like cells', these cells are now referred to as telocytes [46]. Their most characteristic and reliable hallmarks are the ultrastructural features. A typical telocyte shows a small, irregular cell body (average diameter 10 μm), containing a nucleus with a peripheral heterochromatin rim and a scarce cytoplasm with a modest organellular complement. The cell periphery is characterized by a few, very long and thin processes, termed ‘telopodes', whose number determines the shape of the cell body (spindle, triangular or stellate), and lacks a basal lamina (Fig. 3). Telocytes have been described as a normal stromal cell population in the adult and developing heart [47, 48]. Of note, the close relationships between telocytes and cardiomyocytes and the apparent co-orientation of their telopodes with the longitudinal and transverse axes of the cardiomyocytes, which takes place progressively during myocardial development, strongly suggest that telocytes may be crucially involved in the reported property of the cardiac stroma to mould the three-dimensional architecture of the heart [48, 49]. Moreover, telocytes have been postulated to shed microvesicles in the heart interstitium [50], likely working as exosomes to carry informational molecules, such as mRNAs [51]. This could be an additional mechanism whereby telocytes may functionally modulate cardiac muscle cells nearby. In this context, it is noteworthy that cardiac telocytes are particularly sensitive to ischaemia and their three-dimensional network in the normal heart interstitium is destroyed upon myocardial infarction [49]. The disappearance of cardiac telocytes may concur to explain the negligible regenerative ability of the post-infarcted heart. At present, the exact origin and function of cardiac telocytes remains matter of hypothesis and an area for future investigation.

Heart morphogenesis is an intricate process in which cells of different embryonic origin interact to ensure that the heart attains the appropriate size, shape, tissue structure and function. In this process, stromal cells are thought to play an important and unique task, consisting in stimulating the growth and differentiation of cardiac muscle precursors and integrating heart cells into three-dimensional functional assemblies. Multiple observations from *in vitro* and *in vivo* studies concur to support this notion. For instance: (*i*) telocytes have been shown to mediate myocardial compaction from rudimental embryonic trabeculae and regulate ventricular wall organization during mouse heart development [48]; (*ii*) embryonic fibroblasts promote cardiomyocyte settlement in a three-dimensional collagen matrix *in vitro* [52]; (*iii*) cardiac stromal cells selectively express the cardiogenic transcriptional factor GATA-4 [53]; (*iv*) mesenchymal stromal cells stimulate proliferation and differentiation of immature cardiomyocytes [54], an effect involving Notch1/Jagged1-dependent juxtacrine signals [37]. Of note, similar mechanisms appear to remain active in the post-developmental heart, as judged by the findings that: (*i*) in the cardiogenic niches of the adult heart, located in the epicardium close to coronary artery branching [41], telocytes establish close contacts with cardiac stem cells [55]; (*ii*) cardiac stromal cells can promote the dedifferentiation and cell cycle re-entry of adult cardiomyocytes through cell–cell contacts mediated by β-1 integrin [35]. In particular, cardiomyocyte precursors seem to require the physical interaction with cardiac stromal cells for correct recruitment and commitment, a mechanism that may preside over the physiological turnover of the myocardium as well as disease-induced heart repair [56, 57]. From all these data, stromal cell/cardiomyocyte stem cell interactions appear to be necessary for stimulating the regenerative potential of the myocardium. This is a crucial information that can be exploited for the design of effective stem cell–based therapeutic strategies for cardiac regeneration. An additional presumptive role of cardiac stromal cells in cardiac regeneration emerges from the observation that these cells can be reprogrammed *in vitro* to differentiate into cardiomyocytes [59, 60]. However, whether such reprogramming might spontaneously occur in the healing heart remains a matter of speculation, as does the actual contribution of this putative mechanism to *de novo* cardiac muscle formation.

## Repair instead of regeneration: the fee for evolution

Regeneration in lower vertebrates, like fishes and amphibians, is a spectacular phenomenon by which an amputated organ grows back to its original form and recovers its normal function. Among vertebrates, the newt appears to be the most adept at replacing injured organs, including the cardiac ventricles [61, 62]. In these species, regeneration can occur throughout life and consists in wound closure by *de novo* histogenesis that yields the reconstitution of the missing organ. Instead, the vast majority of mammals, including humans, respond to organ injury by a spontaneous repair process, which closes the wound by contraction of its margins and synthesis of scar tissue, which rather hampers regeneration of the injured tissues [63, 64]. Of note, the regenerative ability depends on the peculiar behaviour of mature cells near the site of injury, which are capable of losing their differentiated characteristics and reverting to proliferating stem cells that will later re-differentiate to replace the lost tissues [64]. This mechanism is defined ‘cellular plasticity' [63]. In mammals, embryos can heal wounds spontaneously by regeneration, especially when the injury occurs during early gestation. In foetuses and in newborns, regeneration may take place but quickly vanishes within a few days from birth [65]. In adults, the regenerative ability is permanently lost or limited to superficial epithelial wounds not involving substantially the underlying connective tissue [66]. A comprehensive analysis of the genes and gene regulatory factors involved in cellular plasticity has been reviewed elsewhere [63] and goes beyond the scope of this article. However, it is worth noting that, among the events that play a central role in the regenerative process, there is ECM remodelling [63, 67]. Consistently with this finding, mice of the MRL strain, characterized by a profound capacity for regeneration instead of scarring, display higher MMP activity than their wild-type counterparts [68]. An independent line of evidence indicates that a highly effective biomaterial that can be used to shift an injured adult organ from reparative scarring to at least partial regeneration is a three-dimensional ECM scaffold [64]. Such scaffolds are currently used to induce skin regeneration for plastic and reconstructive surgery purposes [69]. Their mechanism of action seems to consist in the inhibition of the local recruitment of myofibroblasts and hence wound contraction and scarring [66]. Notably, ECM scaffolds display a significantly enhanced regenerative efficacy if endowed with mesenchymal stromal cells [70].

## Conclusions

Taken together, the above notions concur to indicate that stromal cells and ECM have an essential function in the regulation of organ morphogenesis and regeneration. In physiological conditions, stromal cells appear to possess the unique ability to sense the microenvironment, shape a proper three-dimensional scaffold composed of their cell bodies and elongated processes, and stimulate the growth and differentiation of parenchymal precursors to give rise to the complex multi-cellular assembly constituting an organ. In this context, during embryonic development, mesenchymal cells have been found to extend thin filopodes, 1 μm in diameter, interpreted as sensors for spatial information necessary for correct morphogenesis [71]. Such thin filopodes are very similar to the telopodes, the typical processes of telocytes described in numerous developing and adult organs [46]. In pathological conditions, under the guidance of local pro-inflammatory mediators, resident mesenchymal and/or haematopoietic stromal cells recruited from the bloodstream are induced to differentiate into myofibroblasts, which lose the capacity to behave as scaffold moulders and express a fibrogenic phenotype required for prompt deposition of reparatory scar tissue [26]. From an evolutionary viewpoint, repair by scarring could be interpreted as an emergency healing response to injury typical of the most evolved organisms, conceivably directed to preserve survival at the expense of function. Notwithstanding this, a large number of reports in the literature on regenerative medicine indicate that, under appropriate conditions, the original ability of stromal cells to orchestrate organ regeneration can be resumed. Until recently, most research efforts in regenerative medicine have been focused on parenchymal stem cells, their detection and characterization in adult organs, and their actual proliferative and regenerative potential. The new knowledge on the biological properties of stromal cells and their fundamental role as key regulators of the three-dimensional architecture of tissues and organs is rapidly orienting the research towards this fascinating objective.

## Key Points

- Importance of correct three-dimensional organization of multicellular entities
- Role of extracellular matrix in morphogenesis
- Role of stromal cells in morphogenesis
- Different origin of stromal cell populations
- Morpho-functional features of stromal cells in the heart and their possible roles
- Repair instead of regeneration: the fee for evolution
- Conclusions
